# Effect of Game Management on Wild Red-Legged Partridge Abundance

**DOI:** 10.1371/journal.pone.0066671

**Published:** 2013-06-19

**Authors:** Silvia Díaz-Fernández, Beatriz Arroyo, Fabián Casas, Monica Martinez-Haro, Javier Viñuela

**Affiliations:** 1 Instituto de Investigación en Recursos Cinegéticos, IREC (CSIC-UCLM-JCCM), Ciudad Real, Spain; 2 Estación Experimental de Zonas Áridas-CSIC, La Cañada de San Urbano, Almería, Spain; 3 IMAR-Instituto do Mar, Department of Life Sciences, University of Coimbra, Coimbra, Portugal; University of Milan, Italy

## Abstract

The reduction of game and fish populations has increased investment in management practices. Hunting and fishing managers use several tools to maximize harvest. Managers need to know the impact their management has on wild populations. This issue is especially important to improve management efficacy and biodiversity conservation. We used questionnaires and field bird surveys in 48 hunting estates to assess whether red-legged partridge *Alectoris rufa* young/adult ratio and summer abundance were related to the intensity of management (provision of supplementary food and water, predator control and releases of farm-bred partridges), harvest intensity or habitat in Central Spain. We hypothesized that partridge abundance would be higher where management practices were applied more intensively. Variation in young/adult ratio among estates was best explained by habitat, year and some management practices. Density of feeders and water points had a positive relationship with this ratio, while the density of partridges released and magpies controlled were negatively related to it. The variables with greatest relative importance were feeders, releases and year. Variations in post-breeding red-legged partridge abundance among estates were best explained by habitat, year, the same management variables that influenced young/adult ratio, and harvest intensity. Harvest intensity was negatively related to partridge abundance. The other management variables had the same type of relationship with abundance as with young/adult ratio, except magpie control. Variables with greatest relative importance were habitat, feeders, water points, releases and harvest intensity. Our study suggests that management had an overall important effect on post-breeding partridge abundance. However, this effect varied among tools, as some had the desired effect (increase in partridge abundance), whereas others did not or even had a negative relationship (such as release of farm-reared birds) and can be thus considered inefficient or even detrimental. We advise reconsidering their use from both ecological and economical points of view.

## Introduction

The collapse of commercial fisheries and large-scale population declines of other harvested species are particular cases of the current biodiversity crisis that should be dealt within a conceptual framework integrating both ecological and social information [1], [2]. Combining sound ecological knowledge of the harvested species, as well as a proper assessment of the effect of human actions to manage stocks, is essential to optimize long-term exploitation of the harvested resources [3]. Otherwise, incorrect management programs may lead to resource depletion or extinction [4].

Hunting is practiced in many regions throughout the world either for recreation or for subsistence, and it also has an important economic dimension in many areas, sometimes contributing importantly to rural economies [5], [6]. A critical management tool to maintain maximum sustainable harvest on wild game populations is to adaptively adjust harvest through the monitoring of game populations and the establishment of hunting quotas in relation to their abundance [7], [3]. However, robust technical tools for adaptive decision making are scarcely developed or applied yet [4], and management may not be always based on scientific grounds [3]. In fact, many wild game populations have suffered severe declines in recent decades (e.g. [8]), mainly through a combination of environmental factors (such as agriculture intensification or climate change) and overexploitation or incorrect management [4].

Traditional hunting systems have been increasingly altered and sometimes completely replaced by models based on more intensive management. Management focused on increasing or maintaining post-breeding game populations is thus carried out in many areas, particularly when economic interests are strong [9], [10]. The most common management practices applied in Europe to increase small game populations are predator control, habitat management (increase of the quantity or quality of habitats used by game species), species management (provision of supplementary food or water, or medication to decrease parasites), or population supplementation through releases of captive-reared animals [9].

Some of these hunting management practices are controversial and may lead to conflicts between stakeholder groups. For example, predator control has a strong negative connotation for many non-hunters [11] and has been associated in cases to detrimental effects to protected predator populations [12] (and references therein). On the other hand, this technique is frequently considered by hunting managers as necessary to maintain hunting, or at least the economic profitability of this activity, and it may also help population recovery of non-target protected species [13], [14]. Similarly, the use of restocking to increase small game bags has been criticized because of its negative effects on wild game [15], but it may strongly increase hunting yield and economic output of hunting estates in certain cases [16].

Several studies have shown that game management is usually related to higher abundances of target game species (e.g. [17], [18]). This makes it easy to intuitively assume that the pool of management actions applied is useful to increase abundances, but few studies have evaluated the relative efficacy of each practice when applied simultaneously. Such an assessment would help in cost-benefit analyses of different management scenarios to reduce controversies and, ultimately, be able to optimise management decisions for game, biodiversity and society at large.

In Spain, the red-legged partridge *Alectoris rufa* L. is one of the most important small game species numerically and economically [19], [20]. Its importance as a source of economic activity has even grown in recent decades as part of the general Spanish hunting "boom” [21], despite its well recorded, large-scale and deep population decline in the second half of the XXth century [22]. Hunting estates are increasingly managed with costly and specialized techniques [10]. Red-legged partridges are generally more abundant in areas of extensive farmland with high density of edges and a mixture of natural vegetation [23], [24]. Availability of water points influences red-legged partridge distribution [25], but their effect on abundance has not been tested, and there exist few studies about the effects of supplementary food provisioning [26], a very commonly used tool [20], [10]. Predator control, also widely used, is known to be effective to increase game numbers when performed intensively [13], [14], although no studies have been done specifically in Spain where the network of predators (protected and unprotected) is rich and diverse, and their effect on prey populations scarcely known [12]. Finally, negative effects of restocking on wild red-legged partridge populations such as disease spread, changes in population genetic pool or overhunting have been found [27], [28], whereas the effectiveness of small-scale releases to increase hunting yields has been questioned [16].

The intense use and demand of red-legged partridge in Spain [29], and the large geographical spread in management aimed to raise the harvestable stock [20], makes it critical to assess the efficacy and consequences of the management activities to discuss the best management options to maximise long-term economic profitability of the hunting activity and secure conservation of the game species, promoting management favouring ecosystem conservation while minimising the use of techniques that may be harmful.

We studied the relationship between post-breeding partridge abundance (i.e. the huntable stock) and the intensity of various management tools currently applied in hunting estates (food and water supplementation, predator control and restocking), whilst also taking into account two other variables that are known or expected to affect game densities (habitat and harvest levels). We hypothesized that partridge abundance would be higher where management practices were applied more intensively, but we were particularly interested in knowing which contributed more to increase abundance, if applied simultaneously. We discuss our results in terms of their application to improve sustainable exploitation and conservation of a socio-economically important game species with declining wild populations.

## Materials and Methods

We studied 48 hunting estates located in Central Spain, in latitudes ranging from 37.98N to 40.33N and longitudes from 6.48W to 2.11W, ED 1950 (see [16] for a figure). The dominant open landscapes of these estates were characterized by different proportions of cultivated land and natural vegetation (mainly Mediterranean scrub, Table 1). Estate size ranged from 2 to 280 km^2^ (mean±SD = 36.79±54.70; sum of all estates 1765.95 km^2^). Land was mostly privately owned and censuses were carried out with the approval of the person responsible of the game activity within the estate.

**Table 1:** Observed values of analyzed variables in the study estates.

Variable	Mean±SD	Min.	Max.
Summer red-legged partridge abundance (partridges/survey point)	1.24±2.30	0.00	14.00
Arable land (%)	34.71±24.22	0.00	93.02
Vineyards (%)	5.26±9.03	0.00	36.77
Tree crops (%)	8.82±13.10	0.00	41.93
Mediterranean scrub (%)	20.47±23.88	0.00	88.84
Dehesa (%)	13.82±27.96	0.00	100.00
Uncultivated grasslands (%)	12.87±15.03	0.00	66.66
Woodland (%)	1.21+4.42	0.00	26.12
Estate scale Shannon index	0.47±0.23	0.00	0.82
Point scale Simpson index	0.69±0.13	0.49	1.00
Feeders (feeders/km^2^)	3.57±5.41	0.00	25.00
Big water points (big water points/km^2^)	0.88±2.83	0.00	16.52
Small water points (small water points/km^2^)	3.63±5.17	0.00	25.00
Foxes controlled (foxes controlled/yr/km^2^)	1.46±4.04	0.00	25.13
Magpies controlled (magpies controlled/yr/km^2^)	11.54±17.70	0.00	86.21
Partridges released (partridges/km^2^)	14.54±33.57	0.00	188.68
Harvest intensity	0.00±19.16	−30.43	61.23

Game management in the studied estates was mainly aimed to red-legged partridge hunting. We selected privately managed estates, which are the great majority in the region (87% [19]), either with commercial or non-commercial goals, but excluding intensive estates (those with license to release farm-bred partridges without numerical limits throughout the hunting season). Management in intensive estates is qualitatively different from that in other types of estates, and harvest there depends directly on the number of partridges released but not on wild red-legged partridge populations [16], [10]. Studied estates varied largely in the intensity of management performed (as seen in the large SD of means in Table 1, see also [10]).

### Partridge abundance data

We estimated partridge abundance from field observations in each hunting estate, using a point count method [30]. Observers drove along tracks distributed throughout the whole of the estate or, when it was too big, a representative area of the estate stratifying by habitat. Every 700–750 m (exact point depending on visibility of the surrounding area) observers stopped, and partridge numbers and locations were recorded during 10 minutes, using binoculars. Observations took place on early morning (from sunrise to ca. 3 hours later) and in the evenings (3–4 last hours of the day), avoiding the hottest central hours when partridge activity is lowest. Observations were also suspended in case of rain or too windy conditions. Distances from partridges to observer were estimated visually with a precision of 50 m. The number of observation points in each estate was 55±70 (range: 4–425, depending on estate size).

From this information, we calculated a summer partridge abundance index (hereafter partridge abundance) as the sum of recorded partridges within a radius of 300 m at each observation point, divided by the number of observation points monitored in each estate. We selected this simple abundance index instead of e.g. estimating densities from distance sampling, because we were mainly interested in relative abundance estimates that were robust enough to be comparable among hunting estates. Distance sampling estimates need a minimum of observations to reliably estimate detection functions [31], so they lose reliability when abundance is low, as is the case of some of our estates. Additionally, preliminary analyses showed that detection functions mainly depended on observer, as there were significant differences among observers in the estimated distances of observation, particularly in the smaller ranges, whereas there was wide agreement among observers on separating whether observations were within 300 m or beyond. In any case, there was a very good concordance between density estimates based in distance sampling and our abundance estimates based in point counts in estates for which we could estimate densities based on detection functions (r^2^ = 76%, n = 33).

**Figure 1:**
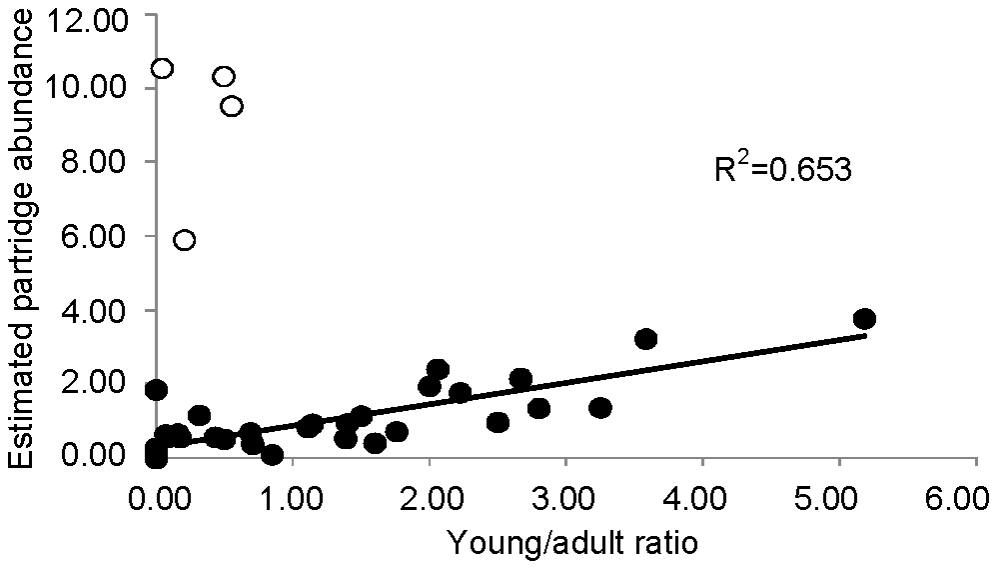
Relationship between young/adult ratio and summer red-legged partridge abundance. Relationship between young/adult ratio and summer red-legged partridge abundance (total partridges observed within 300 m /number of count points). In white, data from the four estates eliminated from analyses (see methods). Also presented is the regression line and r^2^ of the relationship for considered estates.

Additionally, we calculated for each estate the ratio of young to adult partridges observed (hereafter young/adult ratio), when information about partridge age was available (n = 37 estates, in which the proportion of observations of non-aged partridges was lower than 25%). Young/adult ratios can increase with increased young production or with increased adult mortality. Thus, it cannot be considered as directly equivalent to partridge productivity (i.e. the number of young produced by each pair). However, we considered this ratio as indicative of productivity for the goal of this study, as young mortality is much higher than adult mortality during summer months [32], so it seems reasonable to assume that high young/adult ratios mainly reflects young production, rather than high adult mortality. Indeed, our data show that total abundance was positively related to larger young/adult ratios within the sample, supporting this (Fig. 1).

Fieldwork was carried out from mid June to early August, during the red-legged partridge chick rearing period [33]. Specific survey dates of each estate were selected to coincide with the time when most of the cereal had been harvested, in order to maximize visibility, but before farm-bred partridge releases occurred (if they happened at all). In non-intensive estates, releases usually take place as close as possible to the hunting season, i.e., from August to September. From the sample of 48 estates, we excluded from analyses four of those as we had strong suspicions of possible summer releases not reported in the inquiries (high summer abundance with very low young/adult ratio, Fig. 1; and out-of-range annual harvest, see [16]). Surveys were carried out from June 16th to July 31st in 2006 (n = 25) and from June 16th to August 12th in 2009 (n = 19).

### Habitat data

We recorded habitat data during bird surveys as the percentage of each habitat type within a radius of 100 m at each observation point, and then calculated habitat for each estate as the average for all surveyed points. We considered the following habitat variables, defined with functional and management meaning for red-legged partridge [34], [35]: arable land (mostly cultivated with winter cereal or left in annual fallow, the latter usually ploughed during summer; secondarily cultivated with other annual crops), vineyards, tree crops (mainly olive groves, secondarily almond trees, occasionally fig trees), uncultivated grasslands (including old fallow land and uncultivated areas covered by low herbaceous vegetation), Mediterranean scrub (mainly medium-height Mediterranean scrubs, most often *Cistus* sp., *Halimium* sp., *Retama sphaerocarpa*, *Rosmarinus officinalis*, with a strong component of *Quercus coccifera* and holm oak *Quercus ilex*, the latter sometimes achieving full tree height), woodland (pine or eucalyptus plantations, secondarily poplars), or *dehesa* (areas of sparse oak woodland which may be cultivated or grazed underneath). We also calculated habitat Shannon diversity index for each estate from the mean percentage for every habitat category in the estate, and Simpson diversity index [36] as the average of Simpson indexes calculated for each observation point separately. Simpson index is equivalent to the probability that two randomly selected points correspond to the same habitat (maximum diversity if index is cero) and was an indicator of spatial variability in habitat.

### Management and hunting data

We collected management data through face-to-face questionnaires with game managers that voluntarily participated in the study. For each estate, we obtained data on estate size, number of feeders (devices with grain or commercial feed to be consumed by partridges, refilled always during spring and summer, sometimes also in winter), number of artificial water points (see below), number of foxes and magpies annually killed (the two most important predators legally controlled in the area), number of farm-bred partridges annually released and red-legged partridge annual harvest (i.e. number bagged). These data usually corresponded to the hunting season previous to the measure of summer abundances. All variables were expressed per estate surface for analyses.

Artificial water points were of two types: small and large. Small water points, which contain less than 500 l, are water tanks maintaining a constant water level in an external small dish. Large water points are shallow artificial ponds containing more than 500 l of water and covering up more than 100 m^2^. We found that the number of small water points and the number of feeders were highly correlated in the estates (Pearson = 0.87, see Table S1 in Supporting Information), as they are usually placed together. Thus, one of them retains information for both, and we only used the density of artificial feeders in subsequent analyses. Other management variables were not strongly correlated among them (Table S1).

We also obtained an estimate of hunting intensity as the residuals of the linear general model with number of partridges harvested as response variable and partridge abundance as explanatory variable. Our values for number of partridges harvested corresponded to the hunting season previous to the field survey. Between-estate variability in harvest was higher than among-year variability for a given estate, and thus our data represent an adequate estimate of annual harvest for each estate [16].

We lacked data on harvest (and thus hunting intensity) for four of the estates (two of which also lacked information on fox and magpie numbers killed). Two further estates also lacked information on the number of magpies killed. Given that comparisons of AIC between different models are only possible if the same sample is used in all models [37], we eliminated these data for Generalized Linear Models (GLM) analyses. We also carried out analyses with the full (n = 44) sample, replacing missing values for average values from the whole sample. Variables included in the selected models were similar with both data sets, so we present results for the smaller one (more conservative and robust).

### Ethics statement

Following current legislation and ethical rules for human research in Spain, data from questionnaires used in this work does not need approval from an ethics committee, as the data gathered are not related to biological or medical human data [38], and do not “correspond to indentified individuals or individuals possible to be identified” [39]. In fact, they do not correspond to individuals, but to hunting estates, which are not within the current data protection law [39]. We have consulted the Ethics Committee on Human Research of the University of Castilla La Mancha, which consider that the evaluation of such proposal is not within their competence, supporting the statement above.

### Statistical analysis

Analyses were carried out with R 2.13 [40].

We wanted to evaluate which management variables explain summer red-legged partridge abundance while removing the expected effect of other variables (such as habitat). Therefore, we carried out analyses in two steps. First, we tested certain models including habitat variables, based on prior information about habitat needs for the species [23], [34], [41]. At a broad scale, red-legged partridges are known to be more abundant where arable land is the main land use [24], [22]. However, at a smaller scale, highest abundances have been found in those estates with arable land mixed with Mediterranean scrubland, particularly in those with large proportions of both of those habitats [23] [42], and in areas with high farmland habitat diversity [34]. According to this, models we tested included: habitat diversity, and arable land mixed with Mediterranean scrub. These two latter variables were also those that covered on average more than 20% of the surface, and which could attain more than 80% in certain estates (Table 1). We also evaluated whether quadratic variables should also be included, as an optimum of any variable is expected if the ideal landscape includes combinations of both of them. However, no variable fitted a second order polynomial when tested in bi-variate curve-fit analyses (results not presented), so we did not include them. Additionally, as censuses were carried out in two different years, we included “year” as an additional potential variable in all the above models, to account for potential among-year variations in abundance not related to management (Table 2). We evaluated which of those models best explained variations in abundance, according to AICc values. Variables in the selected model were subsequently combined with all management variables. We performed all possible combinations of these independent variables, as all of those models were biologically plausible and we were interested in whether each game management variable alone or in combination with others or with habitat variables could explain partridge abundance variation among estates. We did this with the function dredge (library MuMIn), selected the models with delta AICc <2, and calculated model-averaged parameter estimates for the variables included in those models, as well as their relative importance, calculated as sum of Akaike weights across all the models in the set where that variable occurred [37].

**Table 2:** Results of different GLM models explaining variation in partridge summer abundance in relation to habitat and year (n = 38). k: number of parameters.

	k	AICc	Delta AICc	D-W
a+ms	4	149.59	0.00	2.19
a+ms+yr	5	150.12	0.53	2.19
yr	3	150.46	0.87	2.29
simpson	3	150.52	0.93	2.26
shannon	3	150.56	0.97	2.28
yr+simpson	6	152.69	3.20	2.29
yr+shannon	4	152.79	3.38	2.29
simpson+a	4	152.97	2.93	2.22
yr+a+ms+simpson	6	152.52	3.36	2.29

D-W: Durbin-Watson statistic.

a: arable land; ms: Mediterranean scrub; yr: year; simpson: simpońs index of habitat diversity; shannon: shannońs index of habitat diversity.

An initial exploration of data showed that abundance was positively correlated with young/adult ratio (see results). We evaluated the effect of management variables, the selected habitat variables (Table 2) and year on abundance (using a data set with n = 38 estates for which information on all management variables existed). Additionally, we evaluated the effect of management variables, the selected habitat variables (Table 2) and year on young/adult ratio (using a data set of n = 28 estates for which information on both young/adult ratio and all management variables existed). We assumed that variables affecting abundance that were not included in the model explaining young/adult ratio were variables mostly affecting density of breeders.

Factors affecting variation in response variables (abundance or young/adult ratio) were analysed using GLMs. We fitted response variables to a Gaussian distribution. In all models, we included the variable “number of count points divided by estate area” as a weight, to control for the potential effect of bird census intensity on the abundance estimate. We checked for normality of residuals of the final models. In those, we also calculated the variance inflation factor (VIF, [37]) with the function vif (library HH), considering that a VIF <2 indicates a lack of collinearity among variables within the model [43]; and the Durbin-Watson statistic with the function dwt (library car) to check for autocorrelation of residuals [44].

## Results

Summer red-legged partridge abundance was linearly and positively related to young/adult ratio in our sample of hunting estates (n = 33), the latter explaining 65% of the variance in summer abundance (Fig. 1).

Of all models tested to explain temporal and habitat variations in partridge abundance, the best model included Mediterranean scrub and arable land. Within delta AICc values <2 were also those models including year, or estimates of landscape diversity. However, the combinations of arable land, Mediterranean scrub, landscape diversity and year were much worse (in terms of AICc values) (Table 3). Of the best models (those with delta AICc values <2), we thus chose the one including year, arable land and Mediterranean scrub as the one more biologically meaningful among the equivalent ones.

**Table 3:** Results of GLM models (those models with delta AICc <2) explaining variation in partridge summer abundance or young/adult ratio in relation to management and habitat or year.

	k	AICc	Delta AICc	AICweight	D-W
**Young/adult ratio in relation to habitat, year and management (n = 28)**					
f+r	4	96.2	0.00	0.09	2.31
yr	3	96.4	0.17	0.08	2.30
f+r+yr	5	96.6	0.42	0.07	2.43
mg+yr	4	96.9	0.68	0.07	2.34
(null)	2	97.0	0.75	0.06	2.15
f+r+mg+yr	6	97.0	0.81	0.06	2.60
f	3	97.0	0.84	0.06	2.21
w+f+ms	5	97.3	1.13	0.05	2.44
f+r+yr+hi	6	97.4	1.25	0.05	2.35
f+r+hi	5	97.6	1.40	0.05	2.23
f+mg+yr	5	97.6	1.42	0.05	2.48
f+yr	4	97.8	1.55	0.04	2.30
w+f+r	5	97.8	1.58	0.04	2.14
r+yr	4	97.9	1.70	0.04	2.35
w+f	4	98.0	1.75	0.04	2.06
f+r+mg	5	98.0	1.84	0.04	2.55
f+w+hi+ms	6	98.1	1.86	0.04	2.30
f+hi+ms+r	6	98.1	1.87	0.04	2.54
hi+yr	4	98.1	1.92	0.04	2.28
**Abundance in relation to year, habitat and management (n = 38)**					
a+ms+yr+w+f	8	119.74	0.00	0.45	2.24
a+ms+yr+w+f+hi	9	120.10	0. 48	0.36	2.22
a+ms+yr+w+f+hi +fx	11	121.21	1.77	0.19	2.12

k: number of parameters. D-W: Durbin-Watson statistic.

a: arable land; ms: Mediterranean scrub; f: feeders; w: water points; r: releases; hi: harvest intensity; mg: magpie control intensity; fx: fox control intensity; yr: year.

Best models explaining variations in partridge young/adult ratio included one habitat variable (Mediterranean scrub), year (with higher young/adult ratio observed the second than the first study year), and several management variables: the density of feeders, big water points, intensity of release of farm-reared birds, harvest intensity and magpie control intensity (Table 3). Density of feeders and big water points had a positive relationship with young/adult ratio, while the density of partridges released, harvest intensity or magpies controlled were negatively related to young/adult ratio (Table 4). The parameter estimate of magpies controlled included 0 if taking into account the standard deviation. Of all of these variables, the ones with higher relative importance were feeders and releases (Table 4).

**Table 4:** Model-averaged estimates of the direction and magnitude of each effect size, and relative variable importance (RVI).

Variables	Parameter estimates ± SE	RVI
***Young/adult ratio in relation to habitat, management and year*** (n = 28)		
Feeders	0.068±0.036	0.71
Year (2009)	−1.125±0.647	0.50
Releases	−0.010±0.006	0.48
Magpies controlled	−0.012±0.009	0.21
Harvest intensity	−0.013±0.010	0.20
Water points	0.237±0.160	0.17
Mediterranean scrub	0.010±0.006	0.12
***Abundance in relation to habitat, management and year*** (n = 38)		
Mediterranean scrub	0.035±0.08	1.00
Arable	0.020±0.009	1.00
Feeders	0.060±0.031	1.00
Water points	0.476±0.075	1.00
Year (2009)	1.185±0.456	1.00
Harvest intensity	−0.012±0.008	0.36
Foxes controlled	−0.036±0.033	0.19

Best models explaining variations in summer partridge abundance included the same variables explaining partridge young/adult ratio except magpie control intensity, which was substituted by fox control intensity (Table 3). Feeders, water points, harvest intensity, releases and Mediterranean scrub had all the same type of relationship with abundance as with young/adult ratio, and had also high relative importance (Table 4). Year had a negative relationship with abundance (higher abundance observed in the first study year). Fox control intensity was negatively related to abundance, but had a very low relative importance and its parameter estimate also had a large standard deviation related to the mean (Table 4).

We did not detect problems of autocorrelation of residuals in any of the final best models selected (Durbin-Watson statistic  = ∼2, presented in Table 2). VIF for the variables in the selected best models was between 1.00 and 1.61 and thus, we did not find problems of collinearity in these models.

## Discussion

Our study shows that management and habitat have a strong effect in explaining post-breeding abundances. However, this effect varied among management tools, some of them having a positive effect on abundance, others having undetectable or even detrimental effects. These results allow building an open decision framework for managers, which could be applied to optimise both economic objectives and wild game sustainability. We discuss these ideas below.

### Factors affecting partridge abundance

We found an important effect of year on partridge young/adult ratio or abundance, although the relationship was opposite in both variables. Previous studies have highlighted the strong influence of climatic conditions on red-legged partridge young/adult ratio [45], [46], [33], so observed variations may be related to weather conditions affecting differentially young or adult survival.

Both young/adult ratio and total abundance were positively related to the provision of supplementary food or water, and to the availability of Mediterranean scrub, but negatively to releases of farm-reared partridges or to harvest intensity.

Habitat-related factors are considered crucial to determine the distribution and density of the populations of most species [47], including red-legged partridges. In our study, post-breeding abundance and young/adult ratio were higher in those estates with higher proportion of Mediterranean scrub, which coincides with other studies that suggest that a mixture of scrub and agrarian habitat is an optimal habitat for this species [22], [23]. Overall, habitat was less important than management to explain young/adult ratio. It is possible that some characteristics of farmland management not included here have important influence on partridge young/adult ratio [48], [46]. For example, [35] found that field edges in agricultural landscapes or the timing of cereal harvest were crucial for successful breeding. Similarly, [41] found a high spatial association between brood sizes and field edge density and natural vegetation. Our sample size was not large enough to test for interactions between habitat and other management variables, but it could be envisioned that the effect of food supplementation is larger in those habitats with lower proportion of natural food, or that the effect of predator control is only noticeable in degraded farmland habitats, where protective habitat is scarcer. Further studies should clarify this and consider the inclusion of other agricultural management variables, which may improve the value of habitat models in explaining variations in partridge abundance.

In terms of management techniques, we found that provision of supplementary food was the management variable with the strongest positive effect on young/adult ratio (and thus post-breeding abundance). The provision of food and water has been frequently suggested for gamebird population improvement [49], [3], [50] and is commonly used for red-legged partridges [20]. However, its efficacy has been sometimes contested [51]. In fact, it has also been suggested that food and water supplementation could be unnecessary [3] or even have negative consequences, because feeders and water points could enhance disease transmission through higher contact between individuals [52], [53], and could also enhance attraction of predators and poachers [54]. Our results support a positive relationship between food supplementation and red-legged partridge abundance and young/adult ratio, suggesting that food may be limiting in our managed areas. As stated above, if this was the case we should expect interactions between habitat and the influence of feeder density, which would be interesting to test in future studies. Alternatively, and since the density of feeders was correlated to the density of water points, the effect of feeders could be indicating the beneficial effect of water provision, which is also evidenced in the positive effect of provision of big water points.

Positive effects of water availability on survival or population dynamics have been found in other Mediterranean galliformes [55]. Red-legged partridges use water points usually during summer, especially under harsh climatic conditions [56], and spatial distribution in summer is influenced by presence of water troughs [25]. An alternative explanation to the positive relationship between provision of water troughs and density could be that partridges find other important resources around water points, such as green vegetation, insects or cover. One way or other, our results support that water supplementation in Mediterranean habitats is a beneficial management tool for red-legged partridges, despite this species being well adapted to scarcity of water.

Importantly, partridge young/adult ratio (and thus summer abundance) decreased with increasing numbers of farm-reared partridges released. It may be argued that more partridges are released in areas with poor productivities, but in any case, what is clear from our results is that releases are not effectively increasing summer abundances. It may also be argued that the main goal of releases is just to increase partridge bags in the short term and not to increase summer abundance, and thus effectiveness may not be measured in terms of summer abundance. Indeed, harvest intensity had also a negative strong effect on partridge young/adult ratio and summer abundance. At a larger scale, an increase in hunting pressure has also been found to have a significant effect on the population decline observed in Spain since 1970 [22]. This also suggests that a careful adjustment between take and abundance is critical for population sustainability in this game species. Moreover, [16] showed that releases in low densities are not effective to increase harvest either. Both findings lead to the conclusion that farm-partridge releases in small densities are at best ineffective to increase red-legged partridge hunting bags in the short (annual) or medium term in private hunting estates in Central Spain, as has been previously suggested [57], which could increasing the likelihood of overhunting of wild populations [58]. Otherwise, other studies suggest that releases are indeed negatively affecting the viability of wild populations, through spreading parasites or diseases [22], [28], or modifying the gene pool through the presence of breeding hybrids between *A. rufa* and *A. chukar* in wild populations, due to lack of genetic control of released birds [27].

Finally, it is interesting that we did not find an important effect of predator control intensity on partridge summer abundance or young/adult ratio. We found negative relationships between magpie control and young/adult ratio or fox control and abundance, but these variables had a relative low importance compared with other factors, and the parameter estimates had large standard deviations (including zero at least in the case of magpie control). This agrees with the concerns of [59] about the lack of effect that predator control could have in real-life management situations, contrasted to the effectiveness of very intensive control found in experimental situations [60], [14]. An alternative explanation is that predator control effectiveness interacts with other variables, such as habitat type. One of the limitations of our study (which, in any case, reflects also the limitations of the managers themselves) is that we did not evaluate predator abundance, and hence it is not possible to evaluate the effectiveness of predator control on reducing predator abundance in each of the studied estates, which may explain the observed results. However, [46] did not find a relationship between red fox abundance and predation frequency or summer partridge abundance, so our results may indicate that partridges are able to cope with the levels of predation experienced, or that different predators interact preventing effects to be detected. On the other hand, we found positive relationships between the intensity of fox control and the abundance of another farmland bird, the little bustard *Tetrax tetrax* (authors, unpublished data), which suggests that our measured variable has indeed a biological meaning (i.e., that higher levels of predator control are indeed associated to lower predator numbers). Considering the widespread use of predator control in Central Spain (85.2% of small game estates in Castilla-La Mancha [20]), its low effectiveness for partridges, its possible negative effects for protected predators [12] (and references therein), and its possible positive effects for other protected species [13], [14], a cost-benefit evaluation in different contexts may help to optimize benefits in relation to this management tool. Further studies should concentrate in these important issues.

### Implications for decision making in managers

Decision making in conservation and management is frequently done under a great degree of uncertainty, and with multiple objectives (economic, ecologic and social). Managers frequently have to face trade-offs or dilemmas about how and where to invest in management, which tool to promote or in which circumstances. In many cases, these decisions are taken subjectively, or based on general assumptions. In some cases, investment is made in all potential management tools (like is the case of red-legged partridges), without a real evaluation of the relative costs and benefits of each individual tool.

Our results provide critical information to help managers in their decision making, and could be used, if coupled with an evaluation of the economic cost of each tool, to build an open decision framework for managers, which could be applied to optimise both economic objectives and wild game sustainability.

In the case of red-legged partridges in Central Spain, our results indicate that the best strategy to reinforce wild populations would be to concentrate in improving food and water availability, either directly through providing supplementary food and water (as currently done) or indirectly through improving habitat quality, which could be expected to be a more efficient, stable and profitable long-term strategy [3], [35], [41]. In that sense, measures such as maintaining the right percentage of scrub habitats or keeping a proper density of field edges within the agrarian landscapes, would be also a basic measure to increase red-legged partridge abundances, and thus the availability of this singular renewable resource. On the other hand, investing in small-scale re-stocking with farm-bred partridges is inefficient, potentially detrimental, and should be thus limited in most circumstances (both on economic and ecological arguments). Thus, the “management panacea” of releasing farm-bred birds does not seem to be efficient or secure enough to justify its expanding use as a replacement of correct wildlife resource management, as has been suggested for recreational fisheries too [61].

More generally, our results indicate that some game management practices are more efficient than others, and that their joint application may not lead to additive results. Studies identifying the relative importance of individual management tools when applied simultaneously may thus help evaluating the relative economic and conservation value of different managerial scenarios.

## Supporting Information

Table S1 **Spearman correlation coefficients among the management variables considered.**
(DOC)
