# Supplementary material for: Effect of Game Management on Wild Red-Legged Partridge Abundance
Source: PLoS One. 2013 Jun 19;8(6):e66671. doi: 10.1371/journal.pone.0066671 (PMC3686681; doi:10.1371/journal.pone.0066671)
Supplement: Table S1 — Spearman correlation coefficients among the management variables considered. (DOC) [file pone.0066671.s001.doc]

|  | **Feeders** | **Big water troughs** | **Small water troughs** | **Foxes control.** | **Magpies control.** | **Partridges released** | **Harvest intensity** |
| --- | --- | --- | --- | --- | --- | --- | --- |
| **Feeders** | 1.000 | 0.162 | 0.873 | 0.007 | 0.622 | 0.608 | 0.178 |
| **Big water troughs** | 0.162 | 1.000 | 0.295 | 0.233 | 0.080 | -0.247 | 0.042 |
| **Small water troughs** | 0.873 | 0.295 | 1.000 | 0.138 | 0.714 | 0.588 | 0.076 |
| **Foxes controlled** | 0.007 | 0.233 | 0.138 | 1.000 | -0.140 | 0.039 | 0.319 |
| **Magpies controlled** | 0.622 | 0.080 | 0.714 | -0.140 | 1.000 | 0.495 | 0.092 |
| **Partridges released** | 0.608 | -0.247 | 0.588 | 0.039 | 0.495 | 1.000 | 0.042 |
| **Harvest intensity** | 0.178 | 0.042 | 0.076 | 0.319 | 0.092 | 0.042 | 1.000 |
